# Challenges in the management of iliofemoral deep vein thrombosis in a resource limited setting: a case series

**DOI:** 10.11604/pamj.2014.18.254.2569

**Published:** 2014-07-26

**Authors:** Emeka Blessius Kesieme, Peter Okokhere, Sylvester Eluehike, Peter Isabu

**Affiliations:** 1 Department of Surgery, Irrua Specialist Teaching Hospital, PMB 8, Irrua, Edo State, Nigeria; 2 Department of Medicine, Irrua Specialist Teaching Hospital, PMB 8, Irrua, Edo State, Nigeria; 3 Department of Radiology, Irrua Specialist Teaching Hospital, PMB 8, Irrua, Edo State, Nigeria; 4 Department of Obstetrics and Gynaecology, Irrua Specialist Teaching Hospital, PMB 8, Irrua, Edo State, Nigeria

**Keywords:** Iliofemoral deep vein thrombosis, proximal, systemic anticoagulation, thrombectomy

## Abstract

Iliofemoral deep vein thrombosis is a medical emergency associated with pulmonary embolism, severe postthrombotic morbidity and increased rates of recurrence. We present 3 cases of iliofemoral deep vein thrombosis managed in a setting of limited resources. Results of 2-D Ultrasound scan which suggested proximal DVT was confirmed by Doppler ultrasound scan. Patients were all managed by systemic anticoagulation alone. In experienced hands, it is possible to diagnose iliofemoral DVT with 2-D Ultrasound scan and treatment with systemic anticoagulation alone still has a role. However recent studies have proved clearly the superiority of thrombectomy over systemic anticoagulation alone. There is a need to improve the infrastructure and expertise of clinicians managing these conditions in underdeveloped settings to enable them offer the best to their patients.

## Introduction

Iliofemoral deep vein thrombosis (DVT) is a subset of proximal DVT defined by involvement of the common femoral vein (CFV) and or iliac veins irrespective of thrombus involvement in veins below the CFV or above the iliac vein [1]. It is a severe debilitating problem with high risk of pulmonary embolism (PE) and postthrombotic syndrome.

Venous thromboembolism was generally thought to be rare in Africans; however an autopsy study by Sotunmbi PT et al showed a prevalence rate of 2.9% [2] and 78% mortality for patients admitted with PE was noted by Elegbeleye OO and Femi-Pearse D [3]. Hence the recognition and treatment of iliofemoral DVT becomes absolutely important to prevent morbidity and mortality from DVT.

Lack of basic investigative and treatment modalities have limited the care of these patients to systemic anticoagulation therapy alone. However despite adequate systemic anticoagulation which decreases clot propagation and pulmonary embolism, many patients have been found to develop postthrombotic syndrome as a late complication with features of pain, oedema, skin changes and ulceration.

We present 3 cases of acute iliofemoral DVT diagnosed by B-mode, 2-D Ultrasound scan and managed by systemic anticoagulation alone. We have discussed the current management of this condition to highlight the inadequacies and shortcomings in managing these patients in a resource limited environment. This is to express the need for future improvement in patient's management.

## Methods

Between 1st January 2010 and 1st Jan 2013, three (3) patients were treated for iliofemoral deep vein thrombosis at Irrua Specialist Teaching Hospital, Irrua, Edo State, Nigeria. The following data were collected retrospectively: patient's age and sex, clinical features on presentation, results of investigations including ultrasound scan, treatment and outcome. Data were collected from patient's record.

In the absence of colour doppler USS, a Toshiba scanner (B-mode, 2D) Model Justvision 400 with probe frequency of 6.5MHz was used for 2 patients and a Siemens Sonoline S1-400 with a probe frequency of 7.5MHz for one patient. The diagnosis was confirmed by Doppler ultrasound scan in 2 patients and contrast abdominal CT scan in one patient. These investigations were done in another facility. All the patients were placed on subcutaneous enoxaparin 90-180mg and warfarin. They were followed up in the outpatient clinic after discharge.

## Results

Over the 3 year period, we treated 3 male patients with proximal deep vein thrombosis. The age range was between 17-62 years. All the patients presented with sudden painful right lower limb swelling of about one week duration. Two of the patients were on admission prior to the development of symptom. One was being managed for hypertensive heart disease while the other was being managed for respiratory tract infection. The examination finding that was common among all the patients was a swollen right lower limb with differential warmth.

**Figure 1:**
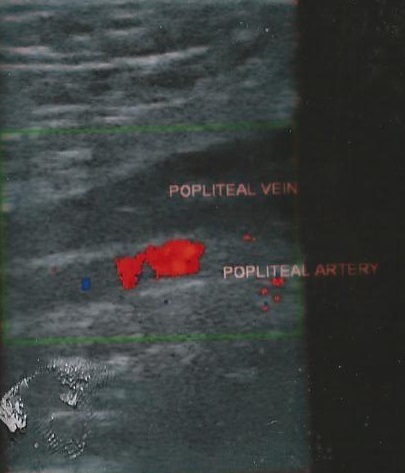
Shows extensive thrombus formation in the popliteal vein

**Figure 2:**
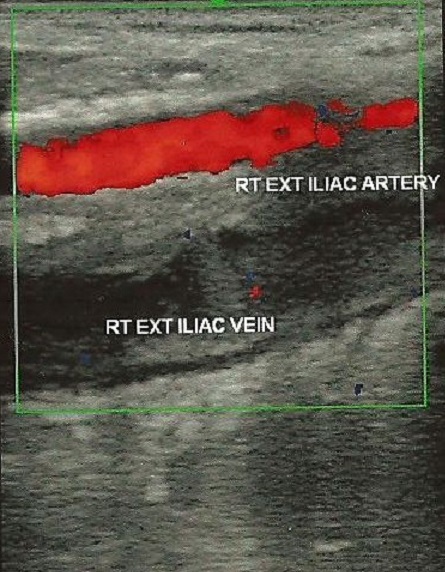
Doppler ultrasound revealed extensive thrombosis involving the iliac veins

A 2-D Ultrasound scan revealed dilatation of the major veins of the lower limb and thrombi were seen in the common femoral vein. Based on the clinical features and the 2-D Ultrasound scan findings, a diagnosis of a proximal DVT was made. In two patients who could afford to pay for a Doppler USS, it was done in another facility and it revealed extensive thrombus formation in the major veins of the left lower limb, extending from the popliteal to the common iliac vein and associated with surrounding soft tissue edema. (Figure 1, Figure 2). Contrast abdominal CT scan done for one of the patients revealed venous stasis involving the left common, internal and external iliac and their branches probably arising from stenosis at its confluence ostium with possibility of associated clot.

They were commenced on subcutaneous enoxaparin 90-120mg daily. Warfarin was introduced at an initial dose of 5mg and was subsequently reduced to 2.5mg. Patient's INR on the last clinic visit were between 2.27 and 2.8. They developed no feature of postthrombotic syndrome or recurrence 8- 15months after discharge.

## Discussion

The clinical symptoms and signs elicited in these 3 cases led to suspicion of and diagnosis of DVT. However the use of a clinical model that standardizes the clinical assessment (combining risk factors and clinical features) and subsequently stratifies patient have been well established, the most commonly recommended model being that developed by Wells and colleagues [4, 5]. The recognized risk factors in these cases were old age, pelvic anatomical abnormality, prolonged immobility, and respiratory tract infection. A strong association between recent respiratory infection and VTE have been described by Clayton et al. They demonstrated an increased risk of DVT in the month following infection and PE in 3 months following infection, both persisting up to a year [6].

D-dimer, a degradation product of cross-linked fibrin, is the best recommended biomarker for the initial assessment of VTE. It is useful in excluding DVT especially in a patient with probability score of less than or equal to 1 [4], however this is not available in our center.

Ultrasonography is an alternative modality for diagnosis of iliofemoral DVT to angiography, being non-invasive and without complication of ionizing radiation. Colour Doppler scan is the technique of choice but in the absence of this; especially in a resource limited setting like ours, B-mode 2D ultrasound scan has been reliable in making diagnosis as seen in our 3 cases. In these cases, non-compressibility of the affected proximal veins of the lower limb using a Toshiba scanner (B-mode, 2D) Model Justvision 400 with probe frequency of 6.5MHz for 2 patients and a Siemens Sonoline S1-400 with a probe frequency of 7.5MHz for one patient. The sonographic findings of dilatation of veins of the affected limb and the presence of a thrombus in the common femoral veins in one case confirmed our suspicion. Diagnosis of iliofemoral DVT was confirmed by Doppler Ultrasound scan in 2 patients who could afford it in an outside facility.

Depending on the level of knowledge, practice and expertise; different modalities of treatment are offered. These include systemic anticoagulation, systemic thrombolysis, catheter directed thrombolysis, and thrombectomy. Systemic anticoagulation was used in all 3 cases.

Systemic anticoagulation has always been the traditional method of treating this condition. However accumulating data now favour early removal of thrombus over systemic anticoagulation alone. A systematic review and metanalysis conducted by Casey et al compared thrombectomy and systemic anticoagulation. Thrombectomy was associated with reduced risk of developing postthrombotic syndrome, venous reflux and a trend for reduction in the risk of venous obstruction [7]. This advantage persisted after 10 years of management [8]. Thrombectomy has been shown to be more effective when delivered within 3 days of onset of symptom [9]. Till date, none of these patients has developed postthrombotic syndrome but this complication is known to occur many years after treatment.

Casy et al found similar results when catheter directed pharmacologic thrombolysis was compared with systemic anticoagulation. There was associated reduction in the risk of postthrombotic syndrome and venous obstruction and a trend for reduction in the risk of venous reflux [7]. Percutaneous transluminal angioplasty and stents have been used in treatment of iliofemoral DVT after thrombus removal. This has been shown to improve quality of life, prevent recurrence of thrombosis, reduce postthrombotic syndromes, and improve ulcer healing [10].

## Conclusion

These three cases have been presented to highlight the inadequacies that physicians in the underdeveloped world encounter while managing proximal deep vein thrombosis. We have also discussed the ideal management of these cases.
